# Emotions and Emotion Regulation in Breast Cancer Survivorship

**DOI:** 10.3390/healthcare4030056

**Published:** 2016-08-10

**Authors:** Claire C. Conley, Brenden T. Bishop, Barbara L. Andersen

**Affiliations:** Department of Psychology, The Ohio State University, 225 Psychology Building, 1835 Neil Avenue, Columbus, OH 43210, USA; bishop.310@osu.edu

**Keywords:** breast cancer, emotion, emotion regulation, quality of life, survivorship, recurrence

## Abstract

Emotional distress in cancer patients is an important outcome; however, emotional experience does not begin and end with emotion generation. Attempts to regulate emotions may lessen their potentially negative effects on physical and psychological well-being. Researchers have called for the study of emotion regulation (ER) in health psychology and psycho-oncology. Thus, this review has three aims. First, we discuss current understandings of emotion and ER across the cancer trajectory, including the principles of ER and methods for its assessment. Second, we present a model for examining the mediating effects of ER on psychosocial outcomes. Third, we “round out” the discussion with an example: new data on the role of ER in recurrent breast cancer. Taken together, these aims illustrate the impact of affective regulatory processes on cancer patients’ long-term outcomes. As survival rates increase, long-term follow-up studies are needed to characterize the dynamic, reciprocal effects of emotion and ER for cancer survivors. Further research on ER may help women with breast cancer better manage the challenges associated with diagnosis and treatment.

## 1. Introduction

The population of cancer survivors continues to grow. In 2012 (the latest year for which information is available), it is estimated that 14.1 million new cancer cases and 8.2 million cancer deaths occurred worldwide [1]. That same year, an estimated 32.6 million people were five-year cancer survivors [2,3]. By 2030, the global burden of cancer is expected to increase exponentially due to the growth and aging of the population [4]. Currently, approximately 1,677,000 new cases of invasive breast cancer are diagnosed annually worldwide [1]. Breast cancer is the second most common cancer overall (11.9% of all new cancer diagnoses) but ranks 5th as cause of death (6.4% of all cancer deaths), leading to a rising number of breast cancer survivors worldwide [1]. The emotional needs of survivors shift as they move through diagnosis and treatment, treatment recovery, and into the years thereafter [5,6,7], making understanding patients’ emotional responses across the timeline important.

Emotional distress has been called the “sixth vital sign” in cancer care [8]. Emotional distress in cancer patients is an important outcome, and when severe it has been associated with reduced treatment compliance [9,10] and elevated risk of disease progression and death [11,12]. In response, authors have called for increased access to supportive resources and organizations (the American Society of Clinical Oncology (ASCO), National Comprehensive Cancer Network (NCCN), and the Institute of Medicine (IOM), among others) have urged screening for emotional distress, and anxiety and depression in particular [13]. However, emotional experience does not begin and end with emotion generation, as there is complementary emotion regulation (ER) in our affective experiences [14,15]. Taken together, the processes of emotion generation and emotion regulation are an essential part of the individual’s subjective cancer experience.

This review has three aims. First, we discuss current understandings of emotion and emotion regulation across the cancer trajectory, including the principles of emotion regulation and methods for its assessment. Second, we present a model for examining the mediating effects of emotion regulation on psychosocial outcomes. Third, we “round out” the discussion with an example: new data on the role of emotion regulation in recurrent breast cancer. Taken together, these aims illustrate the impact of affective regulatory processes on cancer patients’ long-term outcomes.

## 2. Emotional Response to Cancer: What We Know Now

The experience of emotion is a complex and universal human experience. Although affective experiences such as “happiness” and “sadness” may seem straightforward, there is considerable psychological literature dedicated to the understanding of emotions. Emotional experience can be thought of as occurring in two phases. When we experience an event, either positive or negative, emotions are elicited quickly and automatically. In that moment, an emotional stimulus triggers behavioral, physiological, and subjective (e.g., thoughts) responses [16].

### 2.1. What Types of Emotions Do Cancer Patients Experience?

Adjustment to cancer is a process. It includes managing emotional distress [17], and successful adjustment occurs for those patients who are able to regulate their emotional distress. Patients may experience positive emotions, such as hope and gratitude [18,19]. However, many patients may have more difficulty with negative emotions, including anxiety, sadness, anger, guilt, and fear [20]. While all of these emotions can be common reactions to the diagnosis, negative emotions can range from unpleasant to disabling. Extreme, negative emotions may manifest as psychological disorders, such as major depression and situational or generalized anxiety.

Emotional concerns of cancer patients are wide-ranging. A 2013 study reported that the most common concerns of survivors were defining a new sense of normal, managing stress, fear of recurrence, and living with uncertainty [21]. More than 50% of those surveyed reported concerns in four or more of these areas. Though this study was cross-sectional, concerns persisted for patients at different stages in the survivorship trajectory.

### 2.2. How Prevalent Are Negative Emotions among Cancer Patients?

Few studies have directly examined patients’ experiences of negative emotions, but much research has been dedicated to the assessment of psychiatric disorders. These statistics may serve as a proxy for the examination of negative emotions among cancer patients. Rates of clinically significant psychological symptoms in cancer patients often exceed rates in the general population, although estimates of prevalence can vary widely. A few recent studies have begun to narrow the range of these prevalence statistics. Boyes et al. [22] examined a heterogeneous sample of patients and found that the point prevalence of anxiety was 22% and the point prevalence of depression was 13% at six months post-diagnosis. An additional 9% of patients presented with comorbid anxiety and depression. These prevalence rates are consistent with those presented by Linden and colleagues, who examined rates of anxiety and depression in a large (over 10,000) heterogeneous sample [23]. Across types of cancer, 19.0% of patients reported clinical levels of anxiety and 12.9% of patients reported clinical levels of depressive symptoms. A further 22.6% and 16.5% had subclinical symptoms of anxiety and depression, respectively. Notably, patients with breast cancer had significantly higher anxiety and depressive symptoms than other patients, although the authors note that this finding may be attributable to gender differences (e.g., in the general population women exhibit higher rates or clinical depression than men). An additional 9% of patients presented with comorbid anxiety and depression.

### 2.3. Following Treatment, Do Patients’ Emotional Experiences Improve?

There are few behavioral studies of cancer patients that extend any longer than two years post diagnosis. There is considerable evidence showing that, on average, affective symptoms decline during the first year [5], but thereafter, the trajectory is largely unknown.

To our knowledge, four studies have assessed patients’ anxiety through 24 months. While none of these specifically examine anxiety amongst breast cancer patients, their statistics may provide a starting point for considering emotional trajectories in this population. Trudel-Fitzgerald, Gavard, and Ivers [24] assessed a mixed sample of patients (N = 828) with six assessments from post-surgery through 18 months using the Hospital Anxiety and Depression Scale (HADS, [25]). Across time, significant reductions in anxiety occurred, with the greatest change occurring from diagnosis to two months. A similar study by Neilson, Pollard, Boonzaier, Corry, Castle, Smith, Trauer, and Couper [26] assessed head and neck (N = 101) cancer patients. Also using the HADS, there was a significant decline in anxiety from pre- to post-radiotherapy treatment, yet an increase at 18 months. Two other studies, both with gynecologic cancer samples, had similar findings. Mantegna et al. [27] studying cervical cancer patients (N = 227) and their colleagues, Ferrandina et al. [28], studying endometrial cancer patients (N = 132) assessed patients at diagnosis and four additional times through 24 months. For both groups, there was a significant decline in anxiety symptoms as indexed by the HADS from diagnosis to three months, with scores stable thereafter.

Two additional studies provided longer-term data on anxiety responses, although not amongst breast cancer patients specifically. Punnen, Cowan, Dunn, Shuman, Carroll, and Cooperberg [29] surveyed men with prostate cancer (N = 679) treated with radical prostatectomy or not (active surveillance). Using the General Anxiety Disorder Scale 7 (GAD-7, [30]), data was obtained at pre-surgery/baseline, one year, and 1–3 years. Using GAD-7 cutoffs for probable clinically significant anxiety, there were no differences across time or between groups, with moderate to severe levels of anxiety found for less than 5% of the men. Levels of mild anxiety ranged from 15% (baseline/year 1) to 8% (years 1–3). Schroevers, Ranchor, and Sanderman [31] studied a mixed sample of cancer patients (N = 206) at 3 and 15 months and then at 8 years. Using the State-Trait Anxiety Inventory (STAI, [32]), Schroevers found no significant change in anxiety scores across time and, furthermore, no differences with scores from an age-matched sample of healthy individuals. In summary, the data suggest a declining trajectory of anxiety symptoms in the post treatment year (12–24 months) with the greatest change mid or immediately post treatment (3–6 months). The trajectory post 24 months is less clear, although the data suggest the possibility of stability or further declines.

To our knowledge, five studies have provided data on depressive symptoms over time. Unlike studies of anxiety, three of these studies focused exclusively on symptoms among breast cancer patients. Avis, Levine, Naughton, Case, Naftalis, and Van Zee [33] reported low (<4%) rates of moderate to severe depressive symptoms among breast cancer patients (N = 653) 2–3 years post diagnosis. An exceptionally rigorous study was that by Burgess, Cornelius, Love, Graham, Richards, and Ramirez [34] assessing women with breast cancer (N = 170) at least annually over five years. The authors found that the prevalence of depression, anxiety, or both disorders during the first year post-diagnosis was twice that of the general population. However, this number was equivalent to that of the general population after the first year, except for women who experienced a cancer recurrence. Frequent, repeated measurements, such as those included in the Burgess et al. study, may be needed to identify actual adjustment trajectories, as patients frequently shift between clinical and non-clinical groups [35]. Initial assessment of emotional reaction to a diagnosis may not be a reliable predictor of long-term adjustment. Finally, a recent study by Andersen, Goyal, Westbrook, Bishop, and Carson [36] assessed depressive symptoms in breast cancer patients followed continuously for five years. The authors used multiphase linear models to examine phases of change and specific time points of change across the five-year symptom trajectory. Results demonstrated that a steep decline in depressive symptoms occurred by seven months, with stable, low levels thereafter. In summary, the data suggest that the majority of the decline in depressive symptoms occurs soon after diagnosis, within the first 7–12 months; the trajectory thereafter is unclear although a further decline may occur.

Thus, the existing literature on biobehavioral trajectories is limited in size and scope. Though three studies of depressive symptoms examine trajectories among breast cancer patients, there are no corresponding studies of anxiety symptoms. Furthermore, only three psychological outcome studies [31,34,36] provided an additional data point post-24 months. While the majority of cancer patients adjust well following diagnosis, returning to normative levels within 24 months, a large minority reports persistent negative affect [37,38,39,40]. Many authors have attempted to identify the distinct groups of patients with unique adjustment trajectories. Three excellent such studies were those by Henselmans and colleagues [5], Helgeson and colleagues [41], and Andersen and colleagues [37]. First, Henselmans et al. [5] assessed breast cancer survivors within the first year post-diagnosis. The majority experienced no psychological distress (36%) or returned to baseline after completion of active treatment (33%), but smaller groups of survivors became distressed after completion of active treatment (15%) or experienced persistent distress (15%). Second, Helgeson et al. [41] also observed women with breast cancer in an attempt to identify trajectories of functioning, this time through 55 months post-diagnosis. Forty-three percent of this sample demonstrated high and stable mental health over time, while 18% began somewhat lower and improved slightly, 26% evidenced low psychological functioning shortly after diagnosis but showed rapid improvement, and 12% had an immediate and substantial decline in psychological functioning with little to no improvement. Third, Andersen et al. [37] assessed depressive symptoms in breast cancer patients followed continuously for five years. While results demonstrated stable, low levels of depressive symptoms after seven months post-treatment, they also found significant individual differences in the trajectories depressive symptoms from seven months to five years post-treatment. Specifically, some individuals had an increase in depressive symptoms during this time, while others had depressive symptom declines during this time. In short, emotional adjustment trajectories are heterogeneous in populations of cancer patients.

## 3. Emotion Regulation: Basic Principles from Affective Science

### 3.1. What is Emotion Regulation?

Emotion regulation is the process of changing one’s emotions in order to maintain a preferred emotional state [42]. Although there has been considerable debate in the literature as to whether emotion and emotion regulation can be truly parsed out [43,44,45], we take the position that emotion regulation processes can be considered distinct, both theoretically and operationally, from emotion generation processes [14,46]. From this perspective, emotion regulation can be thought of as the bridge between simple emotional experience and conscious emotional expression. In this way, emotion regulation enables emotional homeostasis. Just as the body is able to maintain an internal temperature of 98.6 degrees regardless of the external temperature, the process of emotion regulation allows an individual to maintain a preferred or desired (internal) emotional state following an (external) emotion-eliciting stimulus.

Individuals use a range of ER strategies to alter the valence (positivity versus negativity) or the magnitude of their emotional experiences [47]. Table A1 provides descriptions of commonly used ER strategies. These strategies may be categorized as ‘engagement’ strategies or ‘disengagement’ strategies [48,49,50]. Engagement by cognitive reappraisal and problem solving, for example, aims to change one’s emotions or thoughts following an emotional stimulus. Disengagement strategies, on the other hand, attempt to lessen the impact of an emotion-eliciting event through avoidance or escape, and include techniques such as suppression, cognitive or behavioral avoidance, and substance use.

### 3.2. How Is Emotion Regulation Measured?

Many methods have been developed to assess ER, and presently there is no consensus. Bridges, Denham, and Ganiban [51] raise three foundational issues regarding measurement. First, it is suggested that assessments must target either specific ER strategies or ER as a general construct. Assessment of the “general construct” is often achieved by combining items assessing use of a variety of ER strategies. For example, a broad ER measure may combine items tapping into the separate constructs of cognitive reappraisal, problem solving, avoidance, and suppression. However, combining strategies into broad emotion regulation composites suggests an underlying assumption that use of “more” emotion regulation is better than less (or none at all). If so, one could hypothesize that more regulation would be associated with lower levels of negative emotion, regardless of the ER strategies used. However, more emotion regulation may mean very different things in different contexts or for different individuals [16]. For example, an individual may engage in more ER because he or she is using ineffective or inefficient ER strategies. On the other hand, an individual who engages in more ER may be overly regulated and inhibited—or, as suggested by some, engaging in more ER could truly mean that an individual is appropriately facing an emotion-eliciting situation. In short, more ER may not always be better. Rather than relying on general indices of regulation (e.g., a count of strategies used), Bridges et al. [51] suggest that researchers should identify conceptually distinct categories of behaviors thought to serve similar emotion regulation functions (e.g., adaptive versus maladaptive, approach versus avoidance, engagement versus disengagement, etc.).

Second, assessments must distinguish between the experience of emotion and its regulation. If the goal of the research is to examine relations between emotional experience and emotion regulation, ER measures must avoid overlap between these two constructs. For example, a researcher may be interested in the relationship between emotion regulation and anxiety. He or she might remove “avoidance” items from his or her ER measure, as avoidance is a component of anxiety. If researchers choose to incorporate expressed or experienced emotion into emotion-regulation measures, they should state a theoretical rationale for that choice.

Third, assessments must avoid overlap with outcome measures [52]. Overlap such as this introduces a confound or “third variable” into the research design. When measures contain overlapping items, any correlation between the two may be due to the shared items, rather than a fundamental relation between the two constructs. Let us return to our above example of a researcher interested in the relationship between emotion regulation and anxiety. His or her outcome of interest may be depressive symptoms. This researcher may choose to remove “rumination” items from his or her ER measure, as rumination is a diagnostic consideration for major depressive disorder. Thus, he or she must carefully examine measures assessing both the predictor (emotion regulation) and the outcome (depression) to ensure that any correlation is not due to shared content.

#### 3.2.1. Self-Report Measures

Aldao, Nolen-Hoeksema, and Schweizer [53] provide a review and name over 20 different self-report measures of emotion-regulation, and Brandao and colleagues [54] review 16 emotion regulation measures used in samples of breast cancer patients. We will limit our discussion, however, to a few of those jointly highlighted. These measures are presented in Table A2. Several key features of these measures are presented therein, including constructs assessed, internal validity, and whether or not the scale adheres to recommendations regarding Bridges et al.’s [51] foundational issues in ER assessment. The majority of the scales considered assess ER strategies; only the Courtauld Emotional Control Scale (CECS, [55]) and the Difficulties in Emotion Regulation Scale (DERS, [56]) include total scores reflecting global ER (or in the case of the DERS, emotion dysregulation). Of the seven scales presented, three (the Cognitive Emotion Regulation Questionnaire (CERQ, [57]), the COPE [58,59], and the Emotional Approach Coping Scale (EACS, [60])) have each been shown to have widely varying estimates for subscale internal consistency. Finally, only three scales have minimal content overlap with emotions and outcomes: the CERQ [57], the COPE [58,59], and the Emotion Regulation Questionnaire (ERQ, [61]). Considering these issues, the ERQ has many strengths as a self-report measure of ER: it assesses conceptually distinct ER strategies and does not overlap with other constructs of interest. Despite these strengths, the ERQ is seldom used in studies of breast cancer patients. According to Brandao and colleagues, the most frequently used instrument has been the CECS [54]. Aside from the merits of the scales, there are limitations in using self-report measures to assess individuals’ use of emotion regulation strategies. Authors have questioned the extent to which individuals can accurately identify the emotion-regulation strategy that they use [62]. For example, such self-report measures may require more insight than individuals are capable of. As a result, alternative ER assessment methods have been proposed.

Much of our knowledge of the role of affect regulation in health psychology comes from the study of coping processes. In fact, some coping scales are used as ER measures. The relationship between coping and emotion regulation is, however, complex. Both processes can be conceptualized as ways in which individuals regulate themselves in response to unpleasant or challenging events [15]. In this vein, Gross [16] conceptualizes coping and emotion regulation as different forms of the broader concept of affect regulation. However, the emotion regulation literature distinguishes ER from coping. Emotion regulation is conceptualized as the modification of emotional experience, but coping involves more [63]. This is made clearer by considering that coping processes are often dichotomized, with problem-focused coping and emotion-focused coping being one example. While emotion-focused coping may overlap with emotion regulation, problem-focused strategies would be viewed as coping per se. Thus, emotion regulation overlaps only partially with coping [63].

The distinction between these two constructs is particularly important because there appear to be separate effects of coping and emotion regulation on outcomes, such as quality of life. For example, Karademas and colleagues [64] found that emotion regulation plays a role in determining subjective health that is independent from the impact of illness-focused coping strategies (e.g., problem-focused coping).

#### 3.2.2. Observational Methods

Rather than self-report measures, observation is often used in experimental (laboratory) studies on ER. In them, participants are typically instructed to use a particular ER strategy in response to a standardized, emotion-eliciting stimulus (e.g., a video clip). Researchers then observe the effects of strategy use (e.g., emotional expression, suppression, rumination, etc.) on participants’ subsequent emotions, cognitions, or physiological responses [16,61,65]. Observational methods for the assessment of ER have been extensively reviewed [66,67,68,69]. Experiments provide for strong causal statements about the immediate and short-term effects of ER strategies use, but the particular paradigms used to elicit emotion often suffer from low external validity. Recent evidence suggests some participants are better able than others to follow the instructions of this paradigm [70]. For example, a subject might be shown an emotional video clip and instructed to either express or suppress his or her emotions. While some individuals are easily able to express or suppress emotions on command, others are unable to engage that ER strategy, despite being instructed to do so.

### 3.3. Does Emotion Regulation Affect Psychological Health?

Studies have provided suggestive evidence that use of engagement strategies following an emotion-eliciting stimulus is associated with positive outcomes, including better interpersonal functioning [47], improvement in work performance [71], and reduction in one’s experience of negative affect [72]. On the other hand, data from several studies suggest that disengagement strategies following an emotion-eliciting stimulus are regularly associated with negative psychological outcomes [53,65,73]. More frequent use of disengagement strategies (e.g., denial, suppression) has been related to increased symptoms of depression and anxiety [73], as well as distress that is more intense and longer lasting [53,65,73]. In summary, evidence suggests that disengagement strategies are associated with negative responses, but the extent of beneficial effects for engagement strategies is not clear.

### 3.4. Does Emotion Regulation Affect Physical Health?

There has been a call to study ER in health psychology and in studies of chronic illness in particular [74]. Health threats generate an array of emotions which may impact patients’ well-being [64]. Attempts to regulate emotions may lessen their direct negative effects on physical well-being [74]. Although further research needs to be conducted on the potential mechanisms of these effects, researchers have posited that maladaptive emotion regulation has an impact on biological processes [75]. Because emotion regulation intervenes between stress and health outcomes, it merits consideration both as a significant process and as a point of intervention for reducing the health risks of stress [76].

Engagement strategies have been associated with increased pain tolerance [77] and diminished cardiac reactivity [78]. Alternatively, disengagement strategies have been associated with higher levels of chronic pain [79], increased inflammation [80], higher diastolic blood pressure [75], faster progression of cancer [81,82], and poorer self-reported health [83]. In our review, two studies of emotion regulation among chronic illness populations are of note. First, Van Middendorp et al. [84] examined emotions and ER strategies in women with fibromyalgia and in healthy controls. Their findings demonstrated that women with fibromyalgia utilized the ER strategy of emotional avoidance more frequently than controls. In turn, use of emotional avoidance was related to increased fibromyalgia symptoms, including pain and fatigue. A second study conducted by Stanton and colleagues [85,86] examined the relationship between the ER strategy of emotional expression and physical adjustment to breast cancer. Compared to patients who utilized this strategy infrequently, women high in emotional expression at baseline had fewer medical appointments for cancer-related morbidities and enhanced self-reported physical health and vigor during the next three months. This effect was maintained even when controlling for age, baseline levels of distress, and baseline levels of vigor. Thus, these studies suggest that ER may have a significant impact on physical health outcomes.

## 4. Emotion Regulation in the Context of Cancer: Emerging Evidence

Aldao [87] theorized that emotion regulation processes have four elements: (a) person; (b) situation; (c) individual reactions; and (d) outcomes. We are primarily concerned with the second of these four components: the specific context of emotion regulation. Breast cancer provides a unique setting for the examination of ER, and Ferrer, Green, and Barrett [88] have called for research incorporating cancer and ER. Some evidence demonstrates that ER strategy use may differ in the context of cancer. Compared with healthy women, newly diagnosed breast cancer patients demonstrated higher levels of catastrophizing and acceptance [89]. However, compared with healthy women, this same sample demonstrated lower levels of use of positive reappraisal, self-blame, rumination, positive refocusing, refocusing on planning, and blaming others.

ER has also been linked to patients’ adaptation and well-being [54]. These effects have been found for both disengagement and engagement strategies. Women with breast cancer who frequently used disengagement ER strategies also reported more emotional distress, depressive symptoms, anxiety, lower quality of life, and lower overall emotional well-being [60,85,90,91,92,93,94,95,96]. An interesting study conducted by Leroy and colleagues [97] further highlighted the important role of ER among cancer patients. Using a case-control design, the authors compared the effects of ER difficulties on anxiety and depression symptoms of cancer patients and healthy matched controls. Their results demonstrated that difficulties in emotion regulation were linked to emotional distress in both groups, but accounted for a larger proportion of the variance among cancer patients. Future research with larger samples might provide further support for this unique role of emotion regulation among cancer patients.

Further evidence demonstrates the link between engagement ER strategies and well-being among cancer patients. Peh, Kua, and Mahendran [98] and Sears and colleagues [18] reported that reappraisal is significantly associated with positive emotion, and may be particularly adaptive for patients with low hope. Use of ER strategies such as reappraisal may also predict post-traumatic growth following cancer diagnosis [99,100]. Additional support for the connection between ER and patient-reported outcomes comes from ER-based interventions among patients with cancer. Smyth and Arigo [101] reviewed psychosocial interventions that teach ER skills in the context of health-related outcomes. They concluded that the literature generally supports the use of ER-based interventions to improve well-being in clinical populations. Though intervention research cannot be used to support a causal link between two variables, it does provide further evidence for their relatedness. Finally, the effects of patients’ ER are not limited to their psychological adaptation. ER has also been shown to impact patients’ immune functioning [80,102,103], which in turn have an effect on quality of life and possible cancer progression [104].

## 5. Putting It All Together: A Guiding Emotion Regulation Model

**Figure 1:**
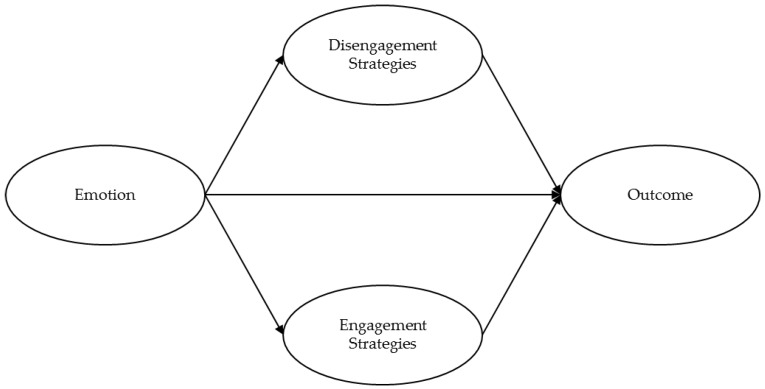
Emotion regulation model.

A model of emotion regulation is presented (Figure 1). It is hypothesized that the affective experiences of an individual have a significant impact on his or her quality of life; however, this impact functions partially through the mechanism of emotion regulation. The emotion regulation techniques utilized by the individual are not replacing negative emotions with positive ones. The individual still experiences the negative emotion, but instead they adjust the dynamics of each emotion and produce adaptive responses to the environment [16,87]. Adaptive responses to the environment lead, in turn, to improved outcomes. In short, an individual first has an affective experience, and the way in which he or she regulates his or her affect influences the relevant outcome.

This perspective has advantages. Firstly, as several authors, including DeSteno and colleagues [74] suggest, the model incorporates separate pathways for the influence of positive and negative emotions, in order to consider the potential synergistic and oppositional effects that may occur in parallel. Secondly, rather than focusing solely on ER as a predictor of adjustment, the model includes meditational pathways. Thirdly, the model typifies a so-called “dual route framework.” That is, life course models suggest that the effects of affect on health may occur by both direct and indirect pathways over time [74], and the model reflects this through the use of partial mediating pathways. Models such as this that allow for partial mediation effects by estimating the direct pathway in addition to the indirect pathway result in a more detailed and nuanced understanding of the potential co-occurring effects.

## 6. New Findings: Emotion Regulation in Recurrent Breast Cancer

Advances have been made in our understanding of affective regulatory processes in individuals who have been diagnosed with cancer, but this research has been primarily limited to the initial cancer diagnosis. Our knowledge of affective regulatory processes in cancer recurrence is inadequate, as cancer recurrence is a qualitatively different and more emotionally intense experience from most stressors. This includes the initial diagnosis of cancer [105,106]. These differences exist on many levels. However, the differences between the experience of an initial breast cancer diagnosis and the experience of recurrence are most pronounced when one considers affect and emotions. An initial breast cancer diagnosis focuses the patient on mobilizing resources, familiarizing oneself with treatments and disease, etc. Instead, recurrence requires mobilization for coping in the long term, i.e., viewing cancer as chronic condition rather than an acute one, coping with maintenance (i.e., continuous) cancer therapy, or having stable (or increasing) symptoms rather than a recovery trajectory. The patient is now faced with a chronic illness, the outcome of which cannot be guaranteed even with adherence to medical treatments and healthy lifestyle. Relinquishing or altering cherished life goals, contending with difficult and changing medical treatments, experiencing effects on intimate relationships, and confronting mortality all are likely to evoke intense emotions [107]. Research demonstrates that patients with recurrent cancer have poorer perceptions of their health, less hope, and more problems in relationships [108].

For these reasons, emotion regulation with recurrence is an important topic to understand, including patients’ emotional responses and the strategies they use to regulate emotional responses. Thus, these data exemplify how ER in the context of cancer might be studied, but they also extend the discussion of ER across the breast cancer trajectory.

### 6.1. Methods

An ER model was tested with patients with recurrent breast cancer. The aim was to evaluate whether emotion regulation strategies mediate the relationship between the emotions of recurrence diagnosis and subsequent quality of life. A single group, repeated measures design was used. Women with recurrent breast cancer (N = 122) were accrued from oncology clinics of a National Cancer Institute-designated Comprehensive Cancer Center affiliated with a large Midwestern university. Patients came from two sources. New, consecutive cases of recurrent breast cancer were accrued to a longitudinal study of coping. Of the 108 women approached, 28 declined and 80 (74%) were accrued. The second source were former participants in a randomized clinical trial (RCT, N = 227) who were diagnosed with Stage II or III breast cancer. Details of informed consent, accrual, and randomization have been published elsewhere [109], and more extensive details regarding study procedures are available from the authors upon request. Women were followed and a subset of patients were later diagnosed with recurrence and approached for accrual to this study on coping with recurrence. Forty-six patients were approached, four (6%) patients declined participation, and thus a total of 42 women participated. Combining the groups, a total of 122 women participated. Preliminary data analyses demonstrated no statistically significant differences in emotion, emotion regulation, or QoL (quality of life) between the groups (all *p*s > 0.11).

The sample was primarily Caucasian (92%), middle aged (*M* = 55, *SD* = 11 years), partnered (70%), and had some college education (69%). On average, the disease-free interval was five years (*SD* = 4 years). At recurrence diagnosis, the majority (67%) had distant metastases rather than loco-regional.

Participants were provided with oral and written informed consent. Following informed consent, a female research assistant assisted women with completing an assessment consisting of self-reports of emotion (The Profile of Mood States Short Form (POMS) [110]; The Center for Epidemiological Studies Depression scale (CES-D), [111]), emotion regulation (Brief COPE, [58]), and quality of life (Medical Outcomes Study 36-Item Short Form Health Survey Mental Health Component Summary (SF-36 MCS), [112]). Follow-up assessments were completed four and 12 months later.

Our hypotheses were twofold. First, higher levels of negative emotions at diagnosis would be negatively associated with later QoL (12 months). Second, ER strategies used in the interim (four months) would mediate the relationship between emotion and QoL. Using the COPE, engagement strategies at four months would be associated with higher QoL at 12 months, and disengagement strategies would be associated with lower QoL.

Structural equation modelling (SEM) was used to test these hypotheses. To that end, a latent variable representing ‘emotion’ was defined using five indicators: POMS subscale scores (tension, depression, anger, and confusion) and CES-D depression. Quality of life (SF-36 MCS) at 12 months was the predicted outcome. To enhance the rigor of the test, analyses controlled for the baseline SF-36 MCS. Sociodemographic, disease, and treatment variables were considered for control. Only variables that were significantly correlated with MCS scores at 12 months were retained.

According to Baron and Kenny [113], a relationship is considered to be mediated when both indirect pathways (in this case, the paths from emotion to regulation strategies and the paths from the regulation strategies to QoL) are significantly different from zero. Thus, the data provided a confirmatory SEM of those variable relations that would be expected if a mediation process occurred.

The model was fit using full information maximum likelihood (FIML) in LISREL version 8.7 (Scientific Software International, Inc., Skokie, IL, USA). Missing data were assumed to be missing at random with an ignorable missing-data mechanism. The FIML estimation approach permits full use of available data. Coefficients were examined for nominal significance (i.e., a coefficient was regarded as having a *p*-value < 0.05 if its magnitude was greater than twice its standard error). Overall fit of the model was evaluated using the Root Mean Square Error of Approximation (RMSEA). In applied research, acceptable RMSEA values are typically those less than or equal to 0.05. However, this is not a rigid cut-off [114].

### 6.2. Results

Of variables considered as controls, only receipt of radiation therapy since diagnosis (0 = no, 1 = yes) was significantly correlated with the SF-36 outcome (*r* = 0.23, *p* = 0.045) and is included. Summary statistics and correlations among measures are reported in Table 1. As anticipated, the sample reported a range of emotional experiences. Mean scores for the POMS subscales of depression and tension were higher than mean scores for the anger and confusion subscales. The mean of the CES-D was below the suggested clinical cut-off of 16; however, the data showed that 34% of patients had scores higher than 16. This suggests that 34% of patients had clinically significant depressive symptoms. Descriptive analyses confirmed that the emotion measures were all positively correlated. Considering emotion regulation, patients reported using engagement strategies more than disengagement strategies. Regarding mental health quality of life (SF-36 MCS), shortly after diagnosis the mean score was 45.30 (*SD* = 10.75), 0.47 standard deviations below the expected population mean of 50. SF-36 MCS significantly improved (*M* = 50.32, *SD* = 9.85) by the 12 month follow-up [*t* = 3.42, *df* = 75, *p* = 0.001].

**Figure 2:**
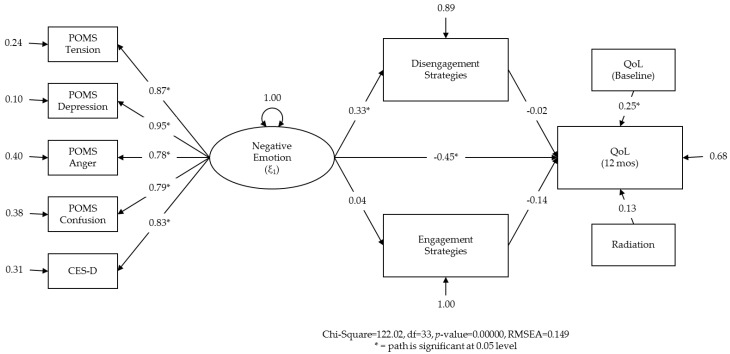
Hypothesized structural equation model testing variable relations that are consistent with parallel mediating effects of engagement and disengagement on the relationship between emotion and quality of life.

The hypothesized structural equation model is shown in Figure 2 with completely standardized solutions. Completely standardized estimates of path coefficients are provided, and the nominal statistical significance of paths is indicated. All factor loading estimates for the latent emotion variable were positive and significant, with the POMS scales and the CES-D all loading onto the latent factor ‘emotion,’ such that higher latent factor scores correspond to higher observed responses on each scale. As predicted, the path from baseline emotion to SF-36 MCS scores at 12 months was significant (standardized β = −0.45, *p* < 0.05). Controlling for QoL at diagnosis, lower levels of negative emotion at diagnosis were related to higher QoL 12 months later. The indirect path from emotion to disengagement was also significant (standardized β = 0.33, *p* < 0.05).

**Figure 3:**
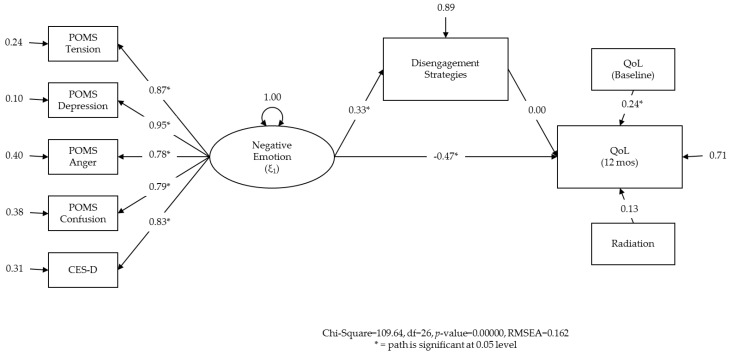
Revised structural equation model testing variable relations that are consistent with the mediating effect of disengagement on the relationship between emotion and quality of life.

The RMSEA was 0.149, suggesting ‘poor fit’ and the need to modify the model. To provide a basis for future research, exploratory analyses were conducted [115]. As indirect paths to engagement were not significant, engagement was removed from the model. In the second model, paths from emotion to all five indicators were again positive and significant (Figure 3). The paths from emotion to QoL and emotion to disengagement were also significant. The RMSEA was similar (0.162). We hypothesized that a model which would use the individual items of the COPE as indicators for the latent factor ‘disengagement’ might enable greater variance to be shared between disengagement and QoL. That is, including the items rather than the subscale score would achieve greater similarity between the measurement model and the theoretical model.

**Figure 4:**
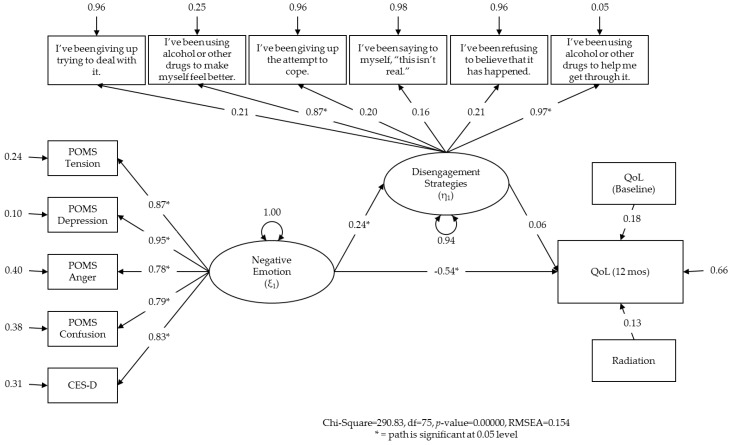
Final structural equation model testing variable relations that are consistent with the mediating effect of disengagement on the relationship between emotion and quality of life. Disengagement is represented as a latent variable.

In the third and final model (using COPE items), paths from emotion to all five indicators were again positive and significant (Figure 4). The paths from emotion to QoL and emotion to disengagement were also significant. The RMSEA was similar (0.154). While SEM did not allow for testing the significance of the indirect effect itself, we can make conclusions regarding the relationships that would be expected in the case of mediation [114]. Given that the path from disengagement to QoL was not significant, these results do not support our hypothesized mediating relationship.

Inspection shows, however, only two paths from disengagement to COPE items were nominally significant (standardized β = 0.87 and 0.97, respectively; *p*s < 0.05). These paths included COPE items 12 (‘I’ve been using alcohol or other drugs to make myself feel better.’) and 25 (‘I’ve been using alcohol or other drugs to help me get through it.’). This suggests that, in this sample, the latent variable ‘disengagement’ reflected the shared variance of two specific COPE items. Interestingly, the other COPE items, reflecting denial and behavioral disengagement, did not significantly contribute to the latent variable ‘disengagement’.

### 6.3. Discussion

This study is among the earliest to examine ER in patients with recurrent cancer. Results further our scant knowledge about the psychological process involved in the emotional adjustment to recurrent cancer, a particularly emotion-eliciting event. Longitudinal data from patients recently diagnosed with breast cancer recurrence provided a strong test of ER as a mechanism in the relationship between emotion and mental health QoL. Two central findings regarding the process and assessment of ER are presented.

First, baseline levels of negative emotions were significantly associated with a decline in QoL at 12 months, as predicted. Since QoL scores in this sample matched the expected population mean by 12 months, an early intervention may provide patients with recurrent breast cancer relief from these time-limited, negative emotions. These results reaffirm that there is a more significant and pressing need among those individuals who experience high levels of negative emotions at diagnosis, because these individuals experience worse QoL outcomes regardless of the ER strategies used. Measures assessing emotions might be used to identify patients most in need of services, consistent with current recommendations for clinical practice [13].

Second, the COPE demonstrated limited utility in assessing ER. This was noteworthy considering the frequent use of the COPE in ER studies [116,117,118,119,120], and may have psychometric and methodological implications. Future research on ER in patients with recurrent cancer should consider other assessment methodologies, as reviewed above.

Taken together with the studies on patients early in the cancer trajectory (i.e., at the time of initial diagnosis) presented in our literature review, new data presented on ER in patients with breast cancer recurrence completes the examination of ER in all phases of cancer survivorship. Thus, affective regulatory processes are important across the entire cancer survivorship trajectory.

## 7. Conclusions

Cancer diagnosis and treatment can elicit strong and varied emotions. Although emotions decrease for the majority of patients, a substantial minority experiences persistent negative emotions. Attempts to regulate these emotions may lessen their direct negative effects on physical and psychological well-being. New data presented provides additional evidence for the impact of emotions and emotion regulation on quality of life for patients with a breast cancer recurrence.

This review allows us to make recommendations for future research. As survival rates increase, long-term follow-up studies are needed to characterize the dynamic, reciprocal effects of emotion and emotion regulation for cancer survivors. Future researchers may also need to consider the methods (and perhaps multiple ones) in assessing emotion regulation among cancer patients.

In conclusion, there is still much to learn about the nature of the relationship between emotion regulation and adaptation to breast cancer. However, it is clear that affective regulatory processes merit significant consideration in both research and clinical practice, due to their intervening role between stress and health outcomes. Further research on emotion regulation may help women with breast cancer better manage the emotional challenges associated with diagnosis and treatment.

## Figures and Tables

**Table 1:** Summary statistics (N = 122) and baseline correlations for emotion, ER, and QoL.

Measure	α	Baseline		Baseline Correlations			4 Months		12 Months	
		Mean (SD)	Range	Engagement	Disengagement	SF-36 MCS	Mean (SD)	Range	Mean (SD)	Range
POMS										
Tension	0.89	6.83 (4.81)	0–23	−0.11	−0.41 *	−0.57 *				
Depression	0.93	6.21 (6.07)	0–27	−0.00	−0.48 *	−0.64 *				
Anger	0.92	4.59 (4.54)	0–28	−0.07	−0.39 *	−0.42 *				
Confusion	0.84	4.43 (3.47)	0–19	−0.09	−0.36 *	−0.57 *				
CES-D	0.92	13.56 (9.60)	0–45	−0.09	−0.38 *	−0.68 *				
Engagement	0.86			1	−0.10 *	−0.08 *	3.02 (1.25)	0.25–5.75		
Disengagement	0.71				1	−0.17 *	0.36 (0.60)	0–2.67		
SF-36 MCS	0.88					1			50.32 (9.85)	24.04–63.08

* Correlation is significant at the 0.01 level (2-tailed).

## Abbreviations

The following abbreviations are used in this manuscript:

ER	emotion regulation
QoL	quality of life

## Appendix

**Table A1:** Descriptions of commonly-used emotion regulation strategies.

ER Domains	Strategy	Description	Examples
Engagement Strategies	Acceptance	Assent to the reality of a situation, without attempting to change it.	“I’ve been accepting the reality of it.”
			“I’ve been learning to live with it.”
	Active coping	Taking active steps to remove/circumvent the emotional stimulus or to minimize its effects.	“I’ve been trying to do something about it.”
			“I’ve been taking action to improve the situation.”
	Cognitive reappraisal	Construing an emotional stimulus in different terms. Also known as reframing.	“I’ve been trying to see it in a different light.”
			“I’ve been looking for the good in the situation.”
	Problem solving	Discovering, analyzing, and finding a solution that best resolves the issue (does NOT include taking active steps toward a solution).	“I’ve been thinking about a strategy for action.”
			“I’ve been thinking about what steps to take.”
	Seeking instrumental support	Seeking advice, assistance, or information from others.	“I’ve been getting help and advice from others.”
Disengagement Strategies	Behavioral avoidance	Reducing one’s effort to deal with the emotional stimulus or attain related goals.	“I’ve been giving up trying to deal with it.”
			“I’ve been giving up the attempt to cope.”
	Cognitive avoidance	Using distraction to prevent oneself from thinking about the emotional stimulus and/or related goals.	“I’ve been trying to take my mind off things.”
			“I’ve been doing something to think about it less.”
	Denial	The refusal to accept reality or fact, acting as if an emotional stimulus did not exist.	“I’ve been thinking that this can’t be real.”
			“I refuse to believe that it has happened.”
	Substance use	Using drugs or alcohol to disengage from or numb an emotional stimulus.	“I’ve been using alcohol or other drugs to make myself feel better.”
	Suppression	Actively trying to put thoughts or feelings about the emotional stimulus out of one’s mind (does NOT include replacing those thoughts with a distractor).	“I tried to put it out of my mind.”
			“I tried to avoid thoughts about it.”

**Table A2:** Descriptions and psychometric properties of self-report measures of emotion regulation used in psycho-oncology.

Scale	Constructs Measured	Number of Items	Internal Consistency	General or Strategies?	Overlap w/Emotions?	Overlap w/Outcomes?
Cognitive Emotion Regulation Questionnaire (CERQ) [57]	Self-blame; Other-blame; Rumination; Catastrophizing; Putting into Perspective; Positive Refocusing; Positive Reappraisal; Acceptance; Refocus on Planning	36	Subscales: 0.59–0.84	Strategies	No	No
COPE Inventory [58,59]	Engagement; Disengagement	60 (Brief = 28)	Subscales: 0.54–0.98	Strategies	No	No
Courtauld Emotional Control Scale (CECS) [55]	Control of Anger; Control of Depressed Mood; Control of Anxiety	21	Subscales: 0.79–0.93; Total score: 0.84–0.95	Both	No	Yes
Difficulties in Emotion Regulation Scale (DERS) [56]	Non-acceptance of Emotions; Difficulties in Goal-directed Behavior; Impulse Control; Lack of Emotional Awareness; Limited Emotion Regulation Strategies; Lack of Emotional Clarity	36	Subscales: 0.80–0.89; Total score: 0.93	Both	Yes	Yes
Emotion Regulation Questionnaire (ERQ) [61]	Cognitive Reappraisal; Expressive Suppression	10	Subscales: 0.68–0.82	Strategies	No	No
Emotional Approach Coping Scale (EACS) [60]	Emotional Expression; Emotional Processing	8	Subscales: 0.32–0.93	Strategies	Yes	No
Ways of Coping Questionnaire (WCQ) [121,122]	Expressing Emotion; Suppression; Avoidance; Wishful Thinking; Problem-Solving; Positive Reappraisal; Escapism	28	Subscales: 0.80–0.81	Strategies	Yes	No
